# Tools for standardized data collection: Speech, Language, and Hearing measurement protocols in the PhenX Toolkit

**DOI:** 10.1111/ahg.12447

**Published:** 2021-09-28

**Authors:** Cynthia C. Morton, Mary L. Marazita, Beate Peter, Mabel L. Rice, Shelly Jo Kraft, Julie Barkmeier‐Kraemer, Carey Balaban, Michael Phillips, Jennifer Schoden, Deborah Maiese, Tabitha Hendershot, Carol M. Hamilton

**Affiliations:** ^1^ Brigham and Women's Hospital, Harvard Medical School, Broad Institute of MIT and Harvard University of Manchester Manchester UK; ^2^ University of Pittsburgh Pittsburgh Pennsylvania USA; ^3^ Arizona State University Phoenix Arizona USA; ^4^ University of Kansas Lawrence Kansas USA; ^5^ Wayne State University Detroit Michigan USA; ^6^ University of Utah Salt Lake City Utah USA; ^7^ RTI International Research Triangle Park North Carolina USA

**Keywords:** common data elements, hearing, language, phenotypes, PhenX Toolkit, speech, standardized measurement protocol

## Abstract

The PhenX Toolkit (https://www.phenxtoolkit.org/) is an online catalog of recommended measurement protocols to facilitate cross‐study analyses for biomedical research. An expert review panel (ERP) reviewed and updated the PhenX Toolkit Speech and Hearing domain to improve the precision and consistency of speech, language, and hearing disorder phenotypes. A three‐member ERP convened in August 2018 to review the measurement protocols in the PhenX Speech and Hearing domain. Aided by three additional experts in voice assessment, vertigo, and stuttering, the ERP updated the 28 protocols to reflect the latest science and technology. ERP recommendations include six new protocols, five updated protocols (from the same source), and one retired protocol. New additions include two voice‐related, three hearing‐related, and two speech‐related protocols. Additions reflect new phone/tablet applications for hearing and language, and clinical evaluations of voice. “Language” was added to the domain name, which is now “Speech, Language, and Hearing,” to represent language‐related protocols. These protocols can facilitate the assessment of speech, language, and hearing in clinical and population research. Common data elements (i.e., use of the same variables across studies) used by geneticists, otolaryngologists, audiologists, speech‐language pathologists, and in other disciplines can lead to cross‐study data integration and increased statistical power when studies are combined.

## 1 INTRODUCTION

The objective of PhenX (consensus measures for Phenotypes and eXposures) is to identify and promote the use of standard measurement protocols that allow for cross‐study analyses and increased statistical power. PhenX, which began in 2006, is funded by the National Human Genome Research Institute (NHGRI), with additional funding from other National Institutes of Health (NIH) Institutes and Centers. During the earlier PhenX process, subject‐matter experts achieved consensus on well‐established measurement protocols that are made available to the public via the PhenX Toolkit (https://www.phenxtoolkit.org/) (Hamilton et al., 2011; Maiese et al., 2013). The PhenX Toolkit currently includes 29 research domains and over 1000 measurement protocols. At this time, the PhenX Toolkit has more than 3500 registered users, has been recommended in more than 400 NIH funding opportunities and notices, and has been cited in over 300 publications. In October 2010, a working group of experts provided content for the Speech and Hearing domain, consisting of 24 protocols associated with 15 measures, that was released into the PhenX Toolkit. To ensure the scientific relevance of these measurement protocols, a three‐member expert review panel (ERP) was assembled to review the existing speech and hearing content in the PhenX Toolkit. During eight months of deliberations, from August 2018 through March 2019, the ERP made recommendations for updating measures and protocols.

## 2 MATERIALS AND METHODS

The ERP was led by a hearing research expert and comprised of speech, language, and hearing experts (roster available at https://www.phenxtoolkit.org/domains/view/200000). To address voice, vertigo, and stuttering, the ERP engaged additional outside experts for recommendations. Members of the ERP presented their assessments and recommendations to the group during six webinars. The ERP solicited feedback on their recommendation via email outreach to more than 2600 PhenX Toolkit users and through personalized communications to investigators in the field. Outreach included information on proposed changes to the protocols.

## 3 RESULTS

The ERP reviewed responses to the email outreach, which were supportive of the proposed updates and additions. The ERP also recommended that the donaim name be changed to Speech, Language, and Hearing to reflect the addition of language‐related protocols. Results of the ERP review and new content added to the domain are described below. In addition to the measurement protocols, the PhenX Toolkit includes data dictionaries, data collection worksheets, and variable mapping with Logical Observation Identifiers Names and Codes (LOINC) to the database of Genotypes and Phenotypes (dbGaP), which will facilitate study implementation and/or analyses. All PhenX protocols are also available in Research Electronic Data Capture (REDCap).

### 3.1 New measurement protocols

1. The hearScreen USA phone/tablet application is based on the Digits in Noise (DIN) test, which is a protocol that can be useful in identifying age‐related hearing loss (hearScreenUSA, 2018–2019). This well‐established protocol has been used in the UK Biobank by 158,000 participants. This screening tool complements the Audiogram hearing test from the National Health and Nutrition Examination Survey (NHANES) protocol in the PhenX Toolkit. The inclusion of this application will assist investigators in detecting hearing loss through new technology.
2. The Auditory‐Perceptual Evaluation of Voice is an assessment of voice quality based on observations of the auditory and perceptual features of an individual. The protocol, the Consensus Auditory‐Perceptual Evaluation of Voice (CAPE‐V), includes sustained phonation of two vowels (/a/ and /i/), reading of six sentences, and spontaneous speaking. The degree of roughness, breathiness, strain, pitch, loudness, and resonance—in addition to the overall severity of voice aberrance—is rated for each speech task using a visual analog scale. The CAPE‐V is designed to take 5 min and can be scored in real time; no instrumentation is required. The protocol is recommended by the American Speech‐Language‐Hearing Association (ASHA) for clinicians and speech‐language pathologists to identify the presence, severity, and characteristics of voice impairments (Kempster et al., 2009).
3. Oral mechanism/Cranial Nerve Examination for Young Children, with the Robbins & Klee Test, is a well‐established protocol since 1987 to identify the potential cause of speech impairments in children (Robbins & Klee, 1987).
4. Phonological awareness uses the syllable repetition task (SRT), a tool to determine how accurately a child or adolescent can repeat nonwords. This screen is suitable for children with or without speech disorders (Shriberg et al., 2009); in addition to problems with phonological awareness, it can detect phonological memory problems (Shriberg et al., 2012).

### 3.2 New protocols added to existing measures

1. A Tinnitus Screener from the National Center for Rehabilitative Auditory Research of the Department of Veteran's Affairs was added to the Tinnitus measure (Henry et al., 2016). This brief questionnaire determines if the respondent has tinnitus, whereas the Tinnitus Handicap Inventory, in the PhenX Toolkit, addresses the personal limitations and quality of life associated with tinnitus.
2. The aging voice index (AVI) for adults aged 65 and older was added to the Voice Impairments measure. This protocol includes questions about how older individuals’ voice affects their daily activities (Etter et al., 2018).

### 3.3 Updates to more current version of the same protocol

1. Grammatical impairments—Grammaticality judgment task was updated with an iPad phone/tablet application of the test, named Grammaggio. For use with children aged 4 years and older, the application produces an audio recording, and the respondent indicates whether the statement is grammatically correct. This type of assessment has been studied in twins and has high heritability estimates as a marker of language impairment relative to age in Australia and in the United Kingdom (Rice et al., 2009).
2. Audiogram hearing test was updated to the 2015–2016 NHANES audiometry test. This hearing test, administered at ages 12 years and older, includes an examination of the outer ear, eardrum mobility test, and acoustic threshold tests at seven frequencies by presenting pure tone signals to each ear via earphones (CDC, 2015–2016).
3. Reading comprehension was updated to the Woodcock–Johnson Tests of Achievement, fourth edition, WJ IV® (2014). This protocol is an interviewer‐administered test for ages 5−70+ years, in which the respondent reads a short passage to identify a missing key word (Schrank et al., 2014).
4. Word decoding was updated to the Test of Word Reading Efficiency–Second Edition, TOWRE‐2 (2012). This protocol assesses the respondent's ability to read real words and nonwords accurately and quickly (Torgesen et al., 2012).
5. Grammatical Impairments – Clinical Evaluation of Language Fundamentals (CELF) was updated to CELF‐5 (2013). These age‐specific protocols assess the respondent's knowledge of grammar (*e.g*., using the appropriate verb) to identify specific categories of speech impairment (Wiig et al., 2013).

### 3.4 Retired protocols

1. The Nonword Repetition measure, which is part of the Comprehensive Test of Phonological Processing (CTOPP) and assesses phonological awareness, was “retired” but remains available via an archived protocols tab on the PhenX Toolkit website (https://www.phenxtoolkit.org/domains/view/200000). The Nonword Repetition measure was replaced with the SRT, which is thought to be a more useful assessment because it is easier to score and can be administered even to children who have speech sound disorders.

### 3.5 Proprietary protocols

Fourteen of the 28 protocols are proprietary and are only available for purchase from companies that sell the tools. The PhenX team obtained copies of the protocols for the purpose of review. The number of proprietary protocols in the domain did not change as a result of ERP review because these protocols are the most well‐established and reliable tools available.

See Table 1 for a complete listing of the measures and protocols in the Speech, Language, and Hearing domain in the PhenX Toolkit.

**TABLE 1:** Speech, Language and Hearing Domain in the PhenX Toolkit

Measure name	Protocol name
**Speech**	
Auditory‐Perceptual Evaluation of Voice (new)	Consensus Auditory‐Perceptual Evaluation of Voice (CAPE‐V)
Oral Mechanism/Cranial Nerve Examination for Young Children (new)	Robbins & Klee Test of Oral and Speech Motor Control
Phonological Awareness (new)	Syllable Repetition Task (SRT)
Phonological Inventory	Goldman‐Fristoe Test of Articulation, Third Edition (GFTA‐3)
Stuttering	Stuttering Severity Instrument, Fourth Edition (SSI‐4)
Voice Impairments (new)	Aging Voice Index
**Language**	
Grammatical Impairments—Clinical Evaluation of Language Fundamentals	Clinical Evaluation of Language Fundamentals, Fifth Edition (CELF‐5)
Grammatical Impairments—Grammaticality Judgment Task	Grammaticality Judgment Task
Grammatical Impairments—Test of Early Grammatical Impairment	Test of Early Grammatical Impairment
Reading Comprehension	Woodcock‐Johnson® Tests of Achievement IV
Vocabulary Assessment	Peabody Picture Vocabulary Test, Fourth Edition (PPVT‐4)
Word Decoding	Test of Word Reading Efficiency, Second Edition (TOWRE 2)
**Hearing**	
Audiogram Hearing Test	2015−2016 National Health and Nutrition Examination Survey (NHANES) Audiometry Protocol
Ear Infections (Otitis Media)	2009−2010 NHANES Audiometry Protocol
Hearing Screener (new)	Digit Triplet Test (in hearScreen USA app)
Personal and Family History of Hearing Loss	Age‐Related Hearing Impairment (ARHI) Questionnaire
Tinnitus	Tinnitus Handicap Inventory
Tinnitus Screener (new)	Tinnitus Screener
Vertigo	Dizziness Handicap Inventory
**Speech and Language**	
Early Childhood Speech and Language Assessment—Ages and Stages Questionnaire	Ages and Stages Questionnaire®, Third Edition (ASQ®‐3)
Early Childhood Speech and Language Assessment—Speech and Language Assessment Scale	Speech and Language Assessment Scale
Family History of Speech and Language Impairment	Family History Questionnaire
**Supplemental Information**	
Instrumental Assessments of Voice (new)	Voice protocols recommended by an American Speech‐Language‐Hearing Association expert panel
Phonological Processes	Khan‐Lewis Phonological Analysis, Third Edition (KLPA‐3)
Pitch Perception Deficit	Distorted Tunes Test (DTT)
Stuttering	Overall Assessment of the Speaker's Experience of Stuttering (OASES™)
Test of Narrative Language	Test of Narrative Language, Second Edition (TNL‐2)
Voice Impairments—Maximum Phonation Time	Maximum Phonation Time Test

## 4 DISCUSSION

The ERP made recommendations for the PhenX Toolkit based on the latest science and availability of established and valid protocols to collect data of interest. Of the 28 protocols in the Speech, Language, and Hearing domain, there were six new protocols, five updated protocols, and one retired protocol. Six protocols are now in supplemental information, which is essentially a second tier of the protocols, and were not omitted because the protocols are useful but not the gold standard.

Measurement protocols in the Speech, Language, and Hearing domain of the PhenX Toolkit cover early childhood to older adulthood. Protocols are provided for assessing family history of speech and language impairment and personal and family history of hearing loss. There are screening tools for tinnitus and age‐related hearing loss. There are protocols for determining whether a person has a voice disorder and others to specify characteristics of the voice disorder. The mode of administration varies by protocol, such as questionnaires for self‐report and interviews; two protocols use software applications for phones or tablets. These measurement protocols are being recommended across disciplines to geneticists and other scientists who are working to distinguish between multiple predisposing risk factors like tinnitus. Use of these protocols will improve the consistency of data collections for speech, language, and hearing phenotypes. For example, phonemic or phonological awareness in dyslexia studies is frequently described as a nebulous cognitive or psychological ability. Phonological does not mean the same thing to some scientists as it does to those working with a linguistic orientation, and this ambiguity is found in the literature. Use of the Phonological Awareness protocol recommended by the ERP could be one way to standardize data collection relevant to language impairments.

As mentioned, the purpose of the PhenX Toolkit is to facilitate the use of available phenotyping tools across displines, which will have several basic science and translational effects. First, easy access to phenotyping tools will directly address the fact that several subspecialties are understudied in terms of genetic risk, influence, and regulation as exampled by developmental stuttering and voice disorders. Second, widely used sets of phenotyping tools will allow direct comparison of results, boosting statistical significance for genetic aspects of communication abilities as a larger number of phenotypic observations will now be available. Third, interdisciplinary use of phenotyping tools will facilitate discovery of shared and unshared biomarkers among disorders that are frequently comorbid, for instance developmental language disorder and dyslexia. Finally, a more comprehensive understanding of genotype–phenotype associations in communication disorders in general has the potential for personalized and proactive clinical translations based on the principles of precision medicine. An example of such a translation is the Babble Boot Camp©, a package of activities and routines currently undergoing clinical trial, designed to prevent or mitigate severe speech and language disorders in infants with predicable genetic risk, in this case a newborn diagnosis of classic galactosemia (Peter et al. in, press; Peter, Potter et al., 2019).

### 4.1 Research gaps

In their February 2019 outreach effort, the ERP asked the research community for help in identifying an established protocol to collect family history of hearing loss for children and for an oral and cranial nerve examination for adults. This content was sought to complement, respectively, the Personal and Family History of Hearing Loss (a protocol for adults and a new measure) and Oral Mechanism/Cranial Nerve Examination for Young Children. To date, no peer‐reviewed literature has been found to fill these measurement voids.

Many different disciplines are involved in communications research. What should researchers interested in inherited factors know about these clinical tests and disorders? Conversely, what should clinicians know about inherited factors, and how can this knowledge be leveraged in clinical practice? A recent survey of over 500 audiologists and speech‐language pathologists showed that knowledge of genetics is considered very important, but actual training and competency in genetics is lacking (Peter, Dougherty et al., 2019).

Many causal genes have already been identified for various forms of hearing impairment (Van Camp et al., 2021) (Cohen et al., 2012). This is due in part to the fact that genetic etiologies of hearing impairment are typically monogenetic in nature. Conversely, genetic influences on disorders of spoken and written language are less understood due to their complexity and heterogenetic influences (Graham et al., 2015). Genetic contributions to balance dysfunction include vestibular disorders and hereditary ataxias (Roman‐Naranjo et al., 2018) (Eppsteiner et al., 2011).

Researchers and clinicians working with children who have cochlear implants are faced with interpreting the large amount of individual variation in language outcomes given the same clinical procedures and hearing acuity levels. Using gene therapy to cure or mitigate some forms of deafness appears to be on the horizon (Omichi et al., 2019). Measures of speech and language development might be used to assess the success or lack thereof in this research. During discussion, the ERP stated that it is time to integrate speech, language, and hearing measures into a range of diseases and conditions studied.

### 4.2 Conclusion

Although speech, language, and hearing disorders are prevalent across all age groups, the level of genetic inquiry is relatively small and overlooked in the medical research community. PhenX Speech, Language and Hearing measurement protocols are being recommended to stimulate interdisciplinary research and promote an interface between research and clinical practice. They are also related to public health. With the advantages of standardized measurements, it is reasonable to believe that the research community will continue to discover inherited components of such communication conditions and to make strides in improving the lives of those affected.

## CONFLICT OF INTEREST

This information or content and conclusions are those of the authors and should not be construed as the official position or policy of, nor should any endorsements be inferred by the NIH, the U.S. Department of Health and Human Services (HHS), or the U.S. Government.

## AUTHOR CONTRIBUTIONS

Deborah Maiese, Tabitha Hendershot, and Carol M. Hamilton provided project guidance and oversight. Michael Phillips and Jennifer Schoden provided project management. CCM, BP, MP, and DM wrote the paper with contributions from all authors.

## Data Availability

Data sharing is not applicable to this article as no new data were created or analyzed in this study.
